# Macronutrient Proportions and Fat Type Impact Ketogenicity and Shape the Circulating Lipidome in Dogs

**DOI:** 10.3390/metabo12070591

**Published:** 2022-06-24

**Authors:** Matthew Irick Jackson

**Affiliations:** Hill’s Pet Nutrition, Topeka, KS 66617, USA; matthew_jackson@hillspet.com; Tel.: +1-985-286-8639

**Keywords:** canine, ketosis, lipidome, macronutrients, metabolome

## Abstract

Many physiological processes including ketogenesis are similar in dogs and humans, but there is little information available on the effect of carbohydrate restriction in dogs. Here, the ketogenicity and serum metabolic profiles of dogs were assessed after they had consumed high carbohydrate (HiCHO); high protein, low carbohydrate (PROT_LoCHO); or high fat, low carbohydrate (FAT_LoCHO) foods. Thirty-six dogs were fed HiCHO for 4 weeks, then randomized to PROT_LoCHO or FAT_LoCHO for 5 weeks. Dogs then crossed over to the other food for an additional 5 weeks. Generally, reduction of dietary carbohydrate by replacement with either protein or fat increased the energy required to maintain body weight, and fat had a greater effect. Postabsorptive energy availability derived mainly from glucose and triglycerides with HiCHO, from gluconeogenic amino acids and fatty acids with PROT_LoCHO, and from fatty acids and β-hydroxybutyrate with FAT_LoCHO. This study demonstrated that the reduction of carbohydrate in canine foods is potentially beneficial to dogs based on improvements in metabolism and supports the use of low-carbohydrate foods as safe and effective for healthy adult dogs.

## 1. Introduction

The proportions of macronutrients, particularly carbohydrate (starches and sugars), that optimize health for companion animal dogs is not known, but is likely dependent on breed [1], activity level [2], health status [3], pregnant or lactating status [4,5,6], and age [7]. Similar to humans, dietary carbohydrate is not nutritionally essential for most canine life stages and was not historically a part of canine food prior to domestication. However, the canine capacity for starch utilization has increased via expanded copy numbers of genes encoding carbohydrate hydrolytic enzymes (AMY2B, MGAM) since the evolutionary divergence of dogs from wolves [8], and in tandem with their co-evolution with humans [9,10]. The prevalence of this additional capacity for starch utilization has continued to expand with human interventional breeding [11]. When sources of dietary starch or whole foods containing starch are fed to dogs, their glycemic response largely parallels that of humans, and it is proposed that the glycemic index developed for humans is relevant to dogs [12]. The levels of carbohydrate-metabolizing enzymes in dogs respond to carbohydrate consumption [13], though not immediately [14]. Further, dietary carbohydrate can alter levels of canine pancreatic proteases and lipases, acting on protein and fat, respectively, [15], which may impact energy availability by increased digestion and absorption [16]. 

When digestible carbohydrate is reduced in foods, those lost calories must be replaced with energy from protein or fat for the food to retain its caloric density. When multiple species, including dogs, consume foods in which carbohydrate is replaced with fat, or, to a more limited extent, protein, a state of nutritional ketosis arises, defined as physiologically elevated circulating β-hydroxybutyrate (βHB) [17]. This state is distinct from pathological ketoacidosis that can accompany diabetes [17,18] or be elicited pharmacologically (e.g., SLGT2 inhibitors) [19]. Reduction of dietary carbohydrate to elicit nutritional ketosis provides health benefits in humans to counteract multiple diseases [20]. Several mechanisms have been proposed to underpin these benefits, including anti-inflammation [21], improved glycemia and lipid metabolism [22], and improved mitochondrial function [23]. Dogs are afflicted with some of the same metabolic diseases as humans, but the degree to which canine disease and metabolic profiles may be impacted by reduction of dietary carbohydrate with alternate macronutrients (e.g., protein or fat) is not known. Reduction in seizures is the most explored therapeutic benefit of low carbohydrate foods in dogs [3]. Improvement of physical performance for sled racing [24] and working dogs [25] has also been reported. 

Many relevant physiological processes, including ketogenesis and regulatory and counter-regulatory hormone responsiveness, are qualitatively similar in dogs and humans. Dogs exhibit similar qualitative characteristics of ketosis as humans in their ratio of acetoacetate:βHB and urinary ketone body output [26], as well as the ability of the brain [27], heart [28], and kidney [29] to utilize ketones (e.g., βHB and acetoacetate) as fuel. When adapted to nutritional ketosis, humans readily withstand decreased availability of circulating energy from glucose [30]; this was similarly shown in dogs by provision of exogenous ketones [31,32]. However, dogs quantitatively differ in their response to, and resolution of, fasting-induced ketogenesis relative to humans. Dogs more slowly achieve ketosis after initiation of fasting, do not achieve as high levels of βHB when ketotic, and also manifest decreases in βHB after carbohydrate re-provision more rapidly than humans [26]. Further, in response to high fat foods and intermittent fasting, dogs do not generate levels of circulating βHB that are considered ketotic in humans [33]. This may be due to dogs’ capacity for sustaining circulating glucose via gluconeogenesis from adipose-sourced, triglyceride-derived glycerol and a lack of pronounced glucagonemia [34], as well as presenting with peripheral musculature highly efficient at utilizing non-esterified fatty acids (NEFA) [35]. Humans consuming low carbohydrate foods typically manifest a reduction in fasting blood glucose [22]. In contrast, in dogs consuming very low carbohydrate foods (<1% of metabolizable energy [ME] by the modified Atwater equation) with various proportions of fat:protein replacing carbohydrate, the fasting blood glucose remained within the standard clinical range for all low carbohydrate foods [36]. Furthermore, there was no reported difference in fasting glucose when dogs consumed foods that were 41% of ME versus 14% of ME as carbohydrate [37]. 

The medium-chain triglycerides (MCT) contain ketogenic medium-chain fatty acids (MCFA) of chain length C8:0 and C10:0 and are readily converted to βHB in the immediate postprandial state. Although there have been documented therapeutic benefits to dogs with seizures when consuming a low carbohydrate food (~3% of ME) [3], due to the inherent resistance of canines to ketosis, it has been proposed that foods intended to induce nutritional ketosis for seizure reduction be supplemented with MCT [38]. Similarly, recommendations for humans vis a vis epilepsy complement a low carbohydrate food with ketogenic MCT [39]. Additionally, it has been observed that MCT hastens the onset of the metabolic switch that underpins a transition into nutritional ketosis [40]; this may be of particular use in ketosis-resistant species such as dogs. In addition to MCT, long-chain unsaturated fats may also increase ketogenicity of foods via activation of the transcription factor peroxisome proliferator-activated receptor (PPAR) α [41], as well as through the intermediacy of their ethanolamide endocannabinoid derivatives oleoylethanolamide [42] and arachidonylethanolamide [43]. Our group has previously reported that a combination of MCT and fish oil increases circulating levels of βHB in companion animal cats consuming a non-ketogenic food [44]. On the other hand, a ketogenic food decreased circulating levels of n3 polyunsaturated fatty acids (PUFA) in an animal model [45]; thus, a ketogenic food may benefit from added n3 PUFA in order to optimally support health and reduce risk of deficiency. 

Most of the available literature regarding dogs and ketosis is derived from fasting experiments (withholding food) or from hormonal interventions. There is little information available on the effect of carbohydrate restriction rather than fasting in dogs, except the aforementioned reports. To our knowledge, no available publications have compared the metabolic effects in dogs of high carbohydrate foods with foods that reduce carbohydrate by replacement with protein versus fat. Some studies [36,37] did not include a carbohydrate replete food in the intervention, and another did not utilize varying levels of protein versus fat or publish their findings except in abstract form [46]. An additional report increased both protein and fat simultaneously, such that individual effects of protein and fat were convoluted [47], and available case studies did not include a high-carbohydrate control [3]. 

The goal of this prospective, randomized study was to assess ketogenicity and macronutrient-metabolism related metabolic profiles of dogs after they had consumed a carbohydrate-rich food, compared with either a food in which carbohydrate energy was replaced with protein or a food in which carbohydrate energy was replaced by fat. Each dog consumed a high-carbohydrate food (HiCHO) for 4 weeks, with fecal and blood collections within the fourth week, and were then randomized to one of the low-carbohydrate foods (PROT_LoCHO or FAT_LoCHO). Following that 5-week period, dogs then crossed over to the other food for a final 5 weeks. Collections were in the fifth week of feeding for both portions of the crossover treatment period. Quantitative clinical serum biochemistries related to macronutrient metabolism and serum fatty acid analysis were assessed. Global metabolomics profiling was also performed on serum for circulating mitochondrial, endoplasmic reticulum, and peroxisomal fatty acid oxidation products along with complex lipid classes including acylcarnitines, endocannabinoids, phospholipids, and sphingolipids.

## 2. Results

### 2.1. Replacment of Carbohydrate in Canine Foods with Protein versus Fat and Impact on Food Macronutrients

Analysis of the nutrient content of the foods is shown in Table 1. The percent of calories from carbohydrate was approximately 375% and 660% higher in the HiCHO food than the PROT_LoCHO and FAT_LoCHO foods, respectively, although the levels of simple sugars were comparable; the difference in carbohydrate levels thus stemmed from starch content rather than from simple sugars. The protein calories were 96% and 112% higher in PROT_LoCHO than FAT_LoCHO and HiCHO, respectively. The FAT_LoCHO food contained 72% and 81% higher fat than the PROT_LoCHO and HiCHO foods, respectively. 

Both LoCHO foods were similar in total dietary fiber percentage of dry matter, while the HiCHO food contained the lowest amount of total dietary fiber and, yet, the highest amount of soluble fiber. Only the FAT_LoCHO food contained an appreciable amount of MCFA and long-chain n3 PUFA (e.g., eicosapentaenoic acid [EPA], docosahexaenoic acid [DHA]) due to the specific inclusion of ingredients harboring these nutrients (fish oil and MCT, respectively). 

### 2.2. Dietary Energy in Relation to Body Weights and Macronutrient Intakes

Dietary ME and nutrient intakes are shown in Table 2. Energy was provided in amounts that were selected to maintain the initial starting BW of the dogs. In the attempt to maintain initial BW, both PROT_LoCHO and FAT_LoCHO foods were required to be fed at significantly higher levels of kcal/(kg BW^0.75^)/day than the HiCHO food (Table 2). The mean ± SE BW at the end of the periods for each food were 9.79 ± 0.11 kg (HiCHO), 9.81 ± 0.10 kg (PROT_LoCHO), and 9.57 ± 0.10 kg (FAT_LoCHO). Despite the increased provision of calories to dogs when they consumed the FAT_LoCHO food, on this food they still had mean BWs that were significantly lower than when they consumed either the HiCHO (*p* < 0.0001) or PROT_LoCHO (*p* < 0.0001) foods. Further, when consuming the PROT_LoCHO food, the dogs maintained the same weight (*p* = 0.994) as when consuming the HiCHO food, despite significantly higher caloric intake (*p* < 0.0001; Table 2).

The intakes of the macronutrients, digestible carbohydrate, protein, and fat were all significantly different among the foods (Table 2). The dogs had approximately 5- and 8-fold higher intake of starch with the HiCHO food than when they consumed the PROT_LoCHO and FAT_LoCHO foods, respectively. When compared to starch intake for the FAT_LoCHO food, the intake of starch from the PROT_LoCHO food was significantly higher. The magnitude of difference in starch content between LoCHO foods was lower than for either LoCHO food versus HiCHO. The intake of total monosaccharide sugars was not different between LoCHO foods, but HiCHO food led to intakes of total sugars that were higher than either LoCHO food by 23–28%. It should be noted that sugars comprised a much smaller proportion of total digestible carbohydrate than starch in all these foods. For protein intakes, the increasing rank order of the three foods was: HiCHO, FAT_LoCHO, PROT_LoCHO. Protein intakes from the PROT_LoCHO food were 102% and 142% higher than from the LoCHO_Fat and HiCHO foods while the FAT_LoCHO food was only 20% higher than the HiCHO food. The increasing rank order of fat intakes on the three foods was: HiCHO, PROT_LoCHO, FAT_LoCHO. Fat intakes from the FAT_LoCHO food were 76–106% higher than from the HiCHO and FAT_LoCHO foods, respectively. Fat intakes on the PROT_LoCHO food were 20% higher than the HiCHO food, concordant with the increased overall caloric intake (Table 2) required to maintain BW. For total dietary fiber as well as insoluble fiber, the increasing rank order of intake was: HiCHO, FAT_LoCHO, PROT_LoCHO, while for soluble fiber it was PROT_LoCHO, FAT_LoCHO, HiCHO. Soluble fiber in each of these foods comprises a much smaller proportion of total dietary fiber than does insoluble fiber. 

### 2.3. Serum Clinical Analyses Reveal That Fat Rather Than Protein Replacement of Carbohydrate Increases Ketogenicity of a Low Carbohydrate Food

Table 3 provides the levels of serum clinical markers relevant to macronutrient metabolism after consuming each of the three experimental foods. Serum glucose levels did not differ among groups. Serum BHB was 43 and 53% higher in the FAT_LoCHO fed dogs than the PROT_LoCHO and HiCHO fed dogs, respectively. Despite being low in carbohydrate (8% of calories as carbohydrate, Table 1), PROT_LoCHO food was not ketogenic in that it failed to elevate circulating ketones above that of the HiCHO food, which had 38% of calories from carbohydrate. Both of the LoCHO foods reduced serum triglycerides 31% below the level observed for the HiCHO food, and were not different from each other. The FAT_LoCHO food led to 15% and 32% increases in total cholesterol relative to the PROT_LoCHO and HiCHO foods, while there was no difference between foods for bilirubin levels (a marker of heme protein catabolism) and no groups presented with lipemia. 

The levels of serum albumin (Table 3) followed the same increasing rank order as that of protein intakes (Table 2), namely: HiCHO, FAT_LoCHO, PROT_LoCHO. The levels of blood urea nitrogen (BUN) also followed this same pattern, with the PROT_LoCHO food resulting in BUN levels that were 46% and 56% higher with PROT_LoCHO relative to FAT_LoCHO and HiCHO, respectively. Serum creatinine values were close in value across foods, with the PROT_LoCHO group higher than HiCHO, but not different than FAT_LoCHO. Creatinine was not different between HiCHO and FAT_LoCHO. 

### 2.4. LoCHO Foods Decrease Serum Amino Acid and Kynurenine Metabolites

The relative levels of serum amino acids and urea from the metabolomics analysis are shown in Table 4 and Table S1. Concordant with the results observed for BUN (Table 3), urea measured by metabolomics was highest when dogs were fed the PROT_LoCHO food; in this assessment, urea was not different between HiCHO and FAT_LoCHO. Serum amino acids were different across foods as a multivariate class (MANOVA *p* < 0.0001). There were 16/21 individual proteogenic amino acids that exhibited a significant food effect by univariate linear mixed model. Overall, the levels of circulating amino acids were present in a pattern opposite to that of protein intake; e.g., the PROT_LoCHO food led to the highest protein intakes and the lowest levels of circulating amino acids. Relative to the HiCHO food, amino acids that were strictly glucogenic were generally decreased following consumption of the PROT_LoCHO food (7/9 of changed amino acids decreased), while 4/5 of those that were dual ketogenic and glucogenic were decreased in the FAT_LoCHO group. With regard to PROT_LoCHO versus FAT_LoCHO, 6/6 differences in strictly glucogenic amino acids were increases with FAT_LoCHO, while 2/2 differences in dual ketogenic and glucogenic amino acids were decreases with FAT_LoCHO. 

The non-proteogenic amino acid taurine did not differ among foods (Table 4). The non-proteogenic methylated glycines observed in the serum metabolomics data (monomethylglycine, dimethylglycine, and trimethylglycine) (Table S1) were all significantly increased following the FAT_LoCHO food; this food contained trimethylglycine as an ingredient, whereas the other two foods did not. Serum glycine itself was not significantly different between FAT_LoCHO and the other foods (Table 4). Thus, provision of dietary trimethylglycine increased all three methylated forms of glycine but did not perturb glycine homeostasis, per se. All three types of methylated glycine were lower following the PROT_LoCHO food relative to the HiCHO food, neither of which foods were supplemented with trimethylglycine. 

Table 5 provides the relative levels of kynurenine pathway metabolites and the differences following consumption of the foods. Kynurenine metabolites were different across foods as a multivariate class (MANOVA *p* < 0.0001). There were 11/12 individual kynurenine-related metabolites that presented a significant effect of food by univariate linear mixed model. Following the FAT_LoCHO food, dogs had the lowest levels of kynurenine metabolites. 

### 2.5. Increased Intake of Fatty Acids Leads to Decreases in those Fatty Acids and Desaturase Activity as Well as Increased Elongase Activity

Table 6 provides the absolute quantitation levels of serum fatty acids for carbon chain lengths of C12 to C22; the C8 and C10 fatty acids are not detected in this analysis. Despite having a significantly higher intake of C12:0, C14:0, C16:0, C16:1 and C18:1 (Table 2), consumption of the FAT_LoCHO food resulted in the lowest or among the lowest levels of these circulating fatty acids (Table 6). Only C18:0, C18:3n3 and the fish oil-derived n3 fatty acids were significantly elevated in serum following the FAT_LoCHO food relative to the HiCHO or PROT_LoCHO foods. The n6/n3 fatty acid molar ratio decreased, while the molar ratio of saturated fatty acids to unsaturated fatty acids (SFA/UFA) increased following consumption of the FAT_LoCHO food relative to either the HiCHO or PROT_LoCHO foods, but the total molar percent of PUFA was not different among foods. 

The summation of quantified fatty acids (Total FA in Table 6) differed among all foods, with the following increasing rank order: HiCHO, PROT_LoCHO, FAT_LoCHO. Circulating total fatty acids were largely orthogonal to circulating triglycerides, with only up to 15% of variation in total fatty acid levels explained by triglyceride levels. The individual group Pearson correlation coefficients for total fatty acids versus triglycerides was: HiCHO (R^2^_adj_ = 0.01; *p* = 0.2503), PROT_LoCHO (R^2^_adj_ = 0.09; *p* = 0.0479), FAT_LoCHO (R^2^_adj_ = 0.15; *p* = 0.0127). Conversely, however, total fatty acid levels were positively and strongly correlated with total cholesterol when dogs were fed any of the three test foods. The individual group correlations for total fatty acids versus cholesterol were all positive and had Pearson correlation coefficients as follows: HiCHO (R^2^_adj_ = 0.87; *p* < 0.0001), PROT_LoCHO (R^2^_adj_ = 0.74; *p* < 0.0001), FAT_LoCHO (R^2^_adj_ = 0.85; *p* < 0.0001). The slopes of the regression lines of total fatty acids versus cholesterol were not different between groups (*p* > 0.05) and averaged around 0.8 mM total fatty acids/mM cholesterol. Triglycerides were also assessed for correlation with cholesterol, but there was not significant correlation for any of the three groups for triglycerides versus cholesterol: HiCHO (R^2^_adj_ = 0.03; *p* = 0.1494), PROT_LoCHO (R^2^_adj_ = 0.04; *p* = 0.1292), FAT_LoCHO (R^2^_adj_ = 0.06; *p* = 0.0793). 

Table 7 provides ratios of fatty acids canonically associated with the enzymatic activity of fatty acid desaturase and elongase enzymes [48]. The FAT_LoCHO food had ratios of fatty acids that were consistent with reduced fatty acid desaturase activity and increased elongase activity relative to both HiCHO and PROT_LoCHO; 4/4 ratios for desaturase activity were lowest in the FAT_LoCHO food and 3/3 ratios for elongase activity were increased. 

### 2.6. Increased Postabsorptive Apparent Circulating Total Energy Availability with Replacement of Dietary Carbohydrate with Fat, but Not Protein

Table 8 shows the mean ± SE values for the total apparent circulating energy as well as for the four individual contributors to the total as a percentage of that total. The available circulating energy after consumption of the FAT_LoCHO food was significantly higher when compared to either HiCHO or PROT_LoCHO, while PROT_LoCHO was higher than HiCHO. The percentage contributions of glucose versus βHB were opposite with the HiCHO versus FAT_LoCHO foods, with glucose forming a higher contribution to total energy for HiCHO than for FAT_LoCHO, and βHB forming a larger contribution to total energy for FAT_LoCHO than for HiCHO. PROT_LoCHO was intermediate between the other two foods for both glucose and βHB. HiCHO derived more of its energy from triglycerides than did either LoCHO food, while adjusted total fatty acids was a larger contributor to total energy for both LoCHO foods than it was for HiCHO. 

### 2.7. Differential Impact of Fat versus Protein Replacement of Carbohydrate on Serum Fatty Acid Energetic Intermediates

Serum α-hydroxy fatty acids (αHFA), products of fatty acid oxidation by endoplasmic reticulum membrane hydroxylases, were different across foods as a multivariate class (MANOVA *p* < 0.0001) (Table S1). There were 10/16 individual αHFA exhibiting a food effect by univariate linear mixed model. Intriguingly, all αHFA with a food effect were 14 carbons or fewer. In line with the provision of MCT in the FAT_LoCHO food, the C4:0, C6:0, and C10:0 (but not C8:0) αHFA were increased in this food relative to either HiCHO or PROT_LoCHO.

Serum β-hydroxy fatty acids (βHFA), products of fatty acids oxidation by mitochondria, were different across foods as a multivariate class (MANOVA *p* < 0.0001) (Table 9 and Table S1). There were 9/11 individual βHFA exhibiting a food effect by univariate linear mixed model. Consumption of the FAT_LoCHO food led to the most altered βHFA. Of these, all 7/7 altered vs PROT_LoCHO were increased by the FAT_LoCHO food and 6/8 different from HiCHO were increased. Of note, the FAT_LoCHO food led to increased βHB in concordance with the measurement of this ketone by quantitative assay (Table 3). Furthermore, the βHFA of MCFA (chain length 6–10) that can be derived from MCT were all increased with the FAT_LoCHO food relative to either other food.

Serum ω-carboxy fatty acids (ωCFA), products of fatty acids oxidation by peroxisomes, were different across foods as a multivariate class (MANOVA *p* < 0.0001) (Table 9 and Table S1). There were 16/19 individual ωCFA exhibiting a food effect by univariate linear mixed model. Similar to the pattern for βHFA (Table 9), consumption of the FAT_LoCHO food had the greatest impact on ωCFA. Where there was a difference for a given ωCFA between FAT_LoCHO and either HiCHO or PROT_LoCHO, the ωCFA was always increased if it was 11 carbons or fewer and decreased if the ωCFA was greater than 11 carbons in length (with the exception of the C20 ωCFA. There was less of an impact for the PROT_LoCHO food compared to HiCHO; only 11/19 differed and, of these, only 3/11 increased, while 8/11 decreased.

Serum mono- and diacylglycerides (MDAG) were different across foods as a multivariate class (MANOVA *p* < 0.0001) (Table S1). There were 5/11 individual MDAG that exhibited a food effect by univariate linear mixed model, and this was driven largely by changes between FAT_LoCHO and the other two foods. Consumption of the FAT_LoCHO food led to changes in 5/11 MDAG relative to HiCHO. The sole decreased MDAG was a diglyceride while all four increased MDAG were monoglycerides. The FAT_LoCHO food further resulted in 4/11 MDAG different from PROT_LoCHO, all with increases of monoglycerides. On the other hand, PROT_LoCHO consumption only altered 1 MDAG (decreased diglyceride) relative to HiCHO. 

Serum acylcarnitines were different across foods as a multivariate class (MANOVA *p* < 0.0001) (Table 10 and Table S1). There were 45/47 individual acylcarnitines that exhibited a food effect by univariate linear mixed model. There was a directional disparity in the response of serum acylcarnitines to the two different LoCHO foods, whereby PROT_LoCHO largely led to decreased acylcarnitines, while the FAT_LoCHO generally increased them. With PROT_LoCHO elevating and FAT_LoCHO decreasing acylcarnitines relative to HiCHO, the starkest differences came between PROT_LoCHO and FAT_LoCHO. Relative to both HiCHO and PROT_LoCHO, the FAT_LoCHO food increased acylcarnitines conjugated to fatty acids derived from added fish oil (22:5n3) and MCT (C8:0, C10:0) dietary fats. Consistent with their low carbohydrate status, both LoCHO foods produced elevations of the carnitine conjugate of βHB relative to the HiCHO food. 

### 2.8. Impact of Carbohydrate Replacement by Fat or Protein on Circulating Levels of Phospholipids and Sphingolipids

Serum choline-containing lysolipids (lysolipids with choline [LysC]) and phospholipids (phosphotidylcholine [PtdC]) were different across foods as a multivariate class (MANOVA *p* < 0.0001) (Table 11 and Table S1). There were 8/10 individual LysC and 24/25 individual PtdC that showed food effects by univariate linear mixed model. The precursor of PtdC via the Kennedy pathway, choline, was increased by the FAT_LoCHO food relative to either other food, though only significantly relative to HiCHO. The PtdC catabolic product glycerophosphorylcholine was increased by both LoCHO foods relative to HiCHO, and increased with FAT_LoCHO relative to PROT_LoCHO. Of the PtdC that increased in FAT_LoCHO relative to PROT_LoCHO, 5/7 contained C18:0. Relative to either food, consumption of the FAT_LoCHO food increased PtdC that contained at least one of the following acyl moieties: C18:0, C18:3n3, C20:4n6, C22:6n3. These acyl moieties were present at disproportionately high levels in the FAT_LoCHO food formulation in relation to the other foods (Table 1) and consumed at disproportionately high levels when dogs ate the FAT_LoCHO food (Table 2). Intriguingly, the PROT_LoCHO food had a specific effect to increase the 1-alkenyl plasmalogens (i.e., those with a 1-enyl group) of PtdC and LysC. Every observed plasmalogen was at a higher level when dogs consumed the PROT_LoCHO food than when eating either other food. 

Serum ethanolamine-containing lysolipids (lysolipids with ethanolamine [LysE]) and phospholipids (phosphatidylethanolamine [PtdE]) were different across foods as a multivariate class (MANOVA *p* < 0.0001) (Table 12 and Table S1). Every one of the individual LysE (8/8) and individual PtdC (16/16) showed food effects by univariate linear mixed model. Neither the PtdE precursor phosphoethanolamine nor the PtdE catabolic product glycerophosphoethanolamine exhibited a food effect overall and the levels of these intermediates was not different between the groups. There were four LysE that differed after consumption of the two LoCHO foods and all of these were lower in the FAT_LoCHO food. Similar to the LysC results, most of the observed LysE 1-alkenyl plasmalogen was increased with the PROT_LoCHO food above the other foods. All four of the PtdE increased by PROT_LoCHO relative to HiCHO were 1-alkenyl plasmalogens. Taken together, all phospholipids and lysolipids that were increased by PROT_LoCHO relative to HiCHO were plasmalogens; there was an apparent greater increase in C16:0-containing plasmalogens than for those with C18:0 (Table S1). Similar to the results for PtdC, the PtdE that increased following FAT_LoCHO relative to HiCHO contained C20:4n6 and C22:6n3 moieties.

Serum sphingolipids and ceramides (together, SPHING) were different across foods as a multivariate class (MANOVA *p* < 0.0001) (Table S1). There were 44/49 SPHING that showed food effects by univariate linear mixed model. Neither sphingosine nor sphingosine-1-phosphate exhibited a food effect overall and the levels of these intermediates were not different among foods. Of the two LoCHO foods, FAT_LoCHO had the greatest impact on SPHING relative to HiCHO. Thus, compared to either other food, consumption of FAT_LoCHO consistently increased SPHING levels. Of particular note, every SPHING containing a C18:0 moiety was increased after FAT_LoCHO consumption relative to either other food; this was not the case for any other SPHING acyl moieties. In contrast, PROT_LoCHO did not consistently elevate SPHING containing the C18:0 moiety. Thus, the specificity, degree, and directionality of change in SPHING relative to HiCHO differed between the two LoCHO foods.

### 2.9. Differential Impact of Carbohydrate Replacement by Fat or Protein on Circulating Levels of Lipid-Derived Neurotransmitters

Lipid-derived N- and O-acylated amino acids and neurotransmitters (together, NAAN) were different across foods as a multivariate class (MANOVA *p* < 0.0001) (Table 13 and Table S1). There were 12/21 individual NAAN that showed food effects by univariate linear mixed model. Relative to consumption of the HiCHO food, the FAT_LoCHO food had a broader impact on NAAN than did PROT_LoCHO and the directionality of effect was largely in the opposite direction for these two LoCHO foods. Further, PROT_LoCHO decreased all 2-acylglycerol-type NAAN and 1 ethanolamide-type NAAN, while FAT_LoCHO affected only a single 2-acylglycerol and none of the ethanolamides. In contrast, consumption of the FAT_LoCHO food led to increases in half of the acylcholines, but PROT_LoCHO only affected one. The differences between LoCHO foods were all increases for FAT_LoCHO relative to PROT_LoCHO. These included acylcholine-type NAAN, acylserine-type NAAN, and a single acyltaurine. Similar to results for other complex lipids (vide infra), the NAAN increased by FAT_LoCHO feeding relative to either HiCHO or PROT_LoCHO most often harbored a C18:0, C20:4n6, C20:5n3 or C22:6n3 acyl moiety. The NAAN detected here only contained acyl moieties of 17 carbons or more, except for a single acyltaurine-type NAAN (hexanoyltaurine, containing a C6:0 acyl moiety). Hexanoyltaurine was elevated by FAT_LoCHO relative to either other food, in concordance with the aforementioned increased C6:0 incorporation into αHFA (Table S1), βHFA (Table 9), and acylcarnitines (Table 10). 

### 2.10. Similar Impact of Carbohydrate Replacement by Fat or Protein on Circulating Primary and Secondary Bile Acids

Primary and secondary bile acids were different across foods as a single multivariate class (MANOVA *p* < 0.0001) (Table 14 and Table S1) and this significance by MANOVA was still present if the primary and secondary bile acids were analyzed as separate multivariate classes. There were 11/16 individual bile acids that showed food effects by univariate linear mixed model (4/6 primary and 7/10 secondary bile acids). Consumption of either LoCHO food exclusively reduced primary and secondary bile acids relative to the HiCHO food; not a single bile acid was higher with either LoCHO food than it was with HiCHO. 

## 3. Discussion

### 3.1. Protein versus Fat Replacement of Carbohydrate Determines Nutritional Ketosis, Dietary Energy Required to Maintain Body Weight and Available Circulating Energy 

How replacement of dietary carbohydrate energy with protein versus fat energy impacts nutritional ketosis in dogs was not previously known. This study sought to compare the effects on nutritional ketosis and macronutrient metabolism of two disparate low-carbohydrate foods (high protein or high fat) to each other and to a high-carbohydrate food (HiCHO). As expected, dogs consumed higher amounts of carbohydrate on the HiCHO food, higher levels of protein on the PROT_LoCHO food, and higher levels of fat on the FAT_LoCHO food. The ketogenic ratio (KR) cutoff, below which a nutritional formulation is no longer predicted to be ketogenic in humans, is >1.5 [49]. No such values for KR threshold are available for canines, and the macronutrient parameter estimates may differ substantially between humans and canines. While strongly emphasizing the anti-ketogenic nature of carbohydrate, the empirically derived KR formula notably considers protein as anti-ketogenic. When the degree of ketogenicity of the foods utilized in the present study was calculated using the KR, only the FAT_LoCHO food had a ketogenic ratio that was above 1.5. According to the macronutrient coefficients in Equation (1) (see Methods), the HiCHO food had the lowest KR due to its carbohydrate content, whereas PROT_LoCHO had an intermediate KR value due to its replacement of carbohydrate by protein rather than fat. After dogs consumed the FAT_LoCHO food, they had higher levels of circulating βHB when compared to either other food, whereas βHB was not different for PROT_LoCHO versus HiCHO. Thus, a canine food with a carbohydrate level below 10% of ME was not ketogenic when the protein was greater than 50% of ME. This demonstrates that the KR formula has some value in predicting the ketogenicity of canine foods since only the food with a KR > 1.5 (FAT_LoCHO) elevated βHB above that of a high carbohydrate food. It should be noted that although levels of dietary ketogenic MCT consumed on the FAT_LoCHO food were much higher than on either other food, samples were collected in the postabsorptive state approximately 20 h after the daily meal, so that observed circulating βHB would be sourced from hepatic conversion of fatty acids arising from lipolysis of stored triglyceride rather than from postprandial dietary fats [50]. 

The amount of food provided daily to the dogs was calculated to maintain BW, and energy offerings were adjusted weekly to account for any changes. In the case of PROT_LoCHO, the dogs required more food to maintain BW than with the HiCHO food; caloric consumption was higher for PROT_LoCHO but BW was not different from HiCHO. When dogs were fed the FAT_LoCHO food, increasing calories were provided weekly in an attempt to maintain BW, such that their daily caloric consumption was higher than for HiCHO. However, even with increased caloric consumption, the dogs’ weights were 2.5% lower with the FAT_LoCHO food than when consuming either other food. Thus, it appears that reduction of dietary carbohydrate to less than 10% ME led to a requirement for increased dietary energy to maintain weight, and that replacement of carbohydrate with fat led to a larger increase in required energy than did carbohydrate replacement with protein. In humans undergoing weight maintenance, low carbohydrate foods also increase the requirement for dietary energy [51], and a rodent model has documented the physiological underpinnings of the effect [52]. Mechanisms relevant to dogs have been proposed whereby this might occur [53], and a biological framework has been proposed that invokes hormonal control of energy balance [54,55]. Whether there is indeed increased energy expenditure when consuming low carbohydrate foods is controversial [56,57,58,59,60,61,62,63,64]. Several studies to date have indicated a statistically significant increase in energy expenditure when humans consume a low carbohydrate food versus a high carbohydrate food [58,63,65,66]. However, it is debated whether the effect of low carbohydrate foods to increase caloric expenditure is transitory [58] and whether the effect size is meaningful enough to result in a physiological difference in weight maintenance or loss [56,63]. In the current study, all foods were analyzed for macronutrients (crude protein, crude fat, total starch, and total sugars), food intakes were measured daily, and BW were measured weekly. Further, with a paired study design the BWs were compared within an individual dog rather than cross-sectionally. Here, a precise assessment of caloric intake could be compared to differences in BW when consuming the two LoCHO and the HiCHO foods in a strictly controlled feeding environment. As a caveat, there was no measurement of energy expenditure in this study, so future studies will need to assess this directly to parse differences in the change to basal metabolic rate versus activity-based energy expenditure. 

### 3.2. Maconutrient-Induced Partitioning of Dietary versus Body Protein towards Amino Acid Gluconeogenesis and Albumin Accretion 

Fasting-based reduction of carbohydrate and protein increases muscle catabolism to amino acids that can serve as gluconeogenic substrates [65], likely by decreasing insulin, which results in increased proteolysis [66], and by increasing glucagon [67], which stimulates the partitioning of amino acids liberated by proteolysis towards gluconeogenesis and urea in dogs [68]. Together, insulin and glucagon dictate glucose availability from gluconeogenesis and glycogenolysis in the postabsorptive dog [69]. The degree to which eucaloric replacement of carbohydrate energy with protein or fat replicates these same effects on insulin and glucagon in dogs is not known. In the current study, dogs were fed at levels that were intended to maintain BW. Diversions from BW versus caloric intake on a single food thus indicate a gross metric of altered energy expenditure. While carbohydrate was restricted, the level of protein energy was highest and intake of protein was most elevated on the PROT_LoCHO food relative to either other food. 

In this study, samples were collected in the postabsorptive state, after dietary protein would have been digested, absorbed, and catabolized to energy-containing substrates and urea, and incorporated into body proteins. At this point in time, there would no longer be any amino acids derived directly from postprandial dietary protein, per se, present in circulation. A previous report demonstrated that normal healthy dogs increase their production of BUN by approximately 25% via increased catabolism of amino acids through the urea cycle when fed a low carbohydrate food containing 46% protein ME versus a non-low carbohydrate food with 20% protein ME [47]. In another report, foods with increasing protein levels and normal carbohydrate levels increased whole-body protein degradation and synthesis in dogs [70]. Thus, in the postabsorptive state reported here, the observed BUN for all groups was derived from proteolysis of body tissues and subsequent catabolism of the resultant amino acids rather than postprandial amino acids. Here, circulating BUN was ~50% higher when dogs consumed the PROT_LoCHO food compared to HiCHO or FAT_LoCHO. Increased BUN may indicate that PROT_LoCHO increased reliance on amino acids for gluconeogenesis to maintain circulating glucose levels. Indeed, fasting glucose was not different between groups. However, the predominant source of that circulating glucose may have differed between dogs fed the HiCHO (hepatic glycogen) versus LoCHO (gluconeogenesis) foods, and perhaps even between the PROT_LoCHO (muscle or hepatic protein-derived amino acids) and FAT_LoCHO (adipose triglyceride-derived glycerol) foods. In support of this interpretation, in the current study 78% of the strictly gluconeogenic circulating amino acids that were different between PROT_LoCHO and HiCHO were decreased by PROT_LoCHO, while 100% of the strictly gluconeogenic amino acids that were different between PROT_LoCHO and FAT_LoCHO were decreased. This supports a model in which PROT_LoCHO feeding leads to a reduced steady state level of amino acids (seen in the metabolomics data) due to increased reliance on amino acid-based gluconeogenesis (seen with clinical BUN). It would appear from this study that consumption of a food in which protein energy replaces carbohydrate energy increases proteolysis of body tissue proteins to amino acids in the postabsorptive state in dogs. This is presumably subservient to the imperative to maintain adequate circulating glucose through gluconeogenesis, and carries, as a consequence, increased production of urea translating into increased BUN. More generally, reduction of dietary carbohydrate may induce partitioning of amino acids into gluconeogenesis, with the protein intake level impacting the magnitude of the effect of carbohydrate reduction by providing additional amino acid substrates for gluconeogenesis.

Circulating albumin was also highest on the PROT_LoCHO food even though all three foods met regulatory body guidance for the level of dietary protein [71,72,73]. Low intake of protein decreases albumin gene expression [74] and alters the synthesis and degradation kinetics of albumin [75], but none of the current foods were deficient in protein to meet the typical criteria for low protein (i.e., below regulatory guidelines). In this study, when dogs were fed the food with the lowest level of protein (25% of ME in HiCHO), their protein intakes were 33–155% greater than the top end of a range of regulatory body minimums and recommendations (2.6–5.0 g/kg^0.75^) for a dog with an activity factor of 1.6 [71,72,73]. Further, FAT_LoCHO fed dogs had protein intakes that were only 20% higher than for HiCHO, leading to an incremental additional intake of protein above that of the regulatory guidelines of 60–207%. Despite these relatively narrow differences in protein intake between HiCHO and FAT_LoCHO, and an incremental increase above regulatory recommendations, FAT_LoCHO increased circulating albumin relative to HiCHO, although it was not increased to the same magnitude as with PROT_LoCHO. Hepatic albumin production may be in competition with glucose production by amino acid gluconeogenesis as both are dependent on amino acid availability from hepatic proteolysis [76]. Taken together, it is concluded that when dietary carbohydrate is reduced, higher protein intakes result in increased postabsorptive period shunting of amino acids derived from tissue proteins into gluconeogenesis and hepatic secretory protein production (e.g., albumin).

### 3.3. Partitioning of Dietary Fat into Fasting Ciculating Lipid Fractions: Energetic Products versus Structural and Signalling Lipids 

The daily intake of total dietary fat and of all measured dietary fatty acids was higher for FAT_LoCHO than for either other food, with the exception that intake of C20:4n6 ARA was the same for FAT_LoCHO and PROT_LoCHO and lowest for HiCHO. In the postprandial state, circulating triglycerides are composed of fatty acids directly derived from the meal rather than from adipose tissue or via de novo lipogenesis. However, in postabsorptive dogs, circulating triglyceride and free fatty acids are endogenously generated rather than directly food-derived [50]. In the postabsorptive state, circulating triglycerides are mostly composed of fatty acids generated by hepatic de novo lipogenesis from carbohydrate precursors [77] or re-esterification of adipose tissue-derived free fatty acids (the triglyceride/fatty acid cycle) [78]. In dogs, free fatty acids increase with the length of time from the last meal [79] and in the postabsorptive state, approximately 15% of circulating free fatty acids are converted by the liver to triglycerides and exported to peripheral tissues, while around 33% is oxidized to ketones (e.g., βHB) and 10% undergo hepatic oxidation [80]. In support of this, in the current study, despite having the highest intake levels of several fatty acids, postabsorptive circulating levels of these fatty acids was lowest in the FAT_LoCHO group. 

Although the dogs were consuming twice as much fat with the FAT_LoCHO food as with HiCHO, the circulating triglyceride levels were approximately one-third less than with HiCHO. Reduction of dietary carbohydrate appeared to drive this effect, since triglyceride levels were one-third less with the PROT_LoCHO food than HiCHO. The fat levels in FAT_LoCHO were well tolerated; there was no difference in the lipemic index between groups. Reduction of dietary carbohydrate also appeared to drive a broad reduction in circulating bile acids as both LoCHO foods manifested decreased levels of primary and secondary bile acids relative to HiCHO, with none increased with the LoCHO foods. There appear to be no published reports describing responses of circulating bile acids to low carbohydrate foods. The level of total fatty acids followed a different pattern from that of triglycerides, whereby the HiCHO group had the lowest levels and the FAT_LoCHO group had the highest levels. The total fatty acids analysis reflects fatty acids derived from multiple structural-type complex lipids, including phospholipids. However, metabolomics data indicated that levels of choline- and ethanolamine-containing phospholipids on balance were lower following both LoCHO foods than the HiCHO food, so the increased total fatty acids observed for the FAT_LoCHO food are not likely to have originated from phospholipids. The increased total fatty acids following the FAT_LoCHO food also would not have been driven by triglycerides since the latter were also decreased. 

Increased availability of circulating energy has been proposed in the postprandial state for humans when consuming a low carbohydrate food relative to a high carbohydrate food [81]. Circulating total potential energy was approximated to assess total apparent circulating energy in postabsorptive dogs in the current study by adding together the relevant caloric equivalents from circulating glucose, βHB, triglycerides, and adjusted total fatty acids (representing both free fatty acids and those from complex lipids such as phospholipids but without triglyceride energy). By this approximation, feeding the FAT_LoCHO food gave the highest level of total potential energy, with PROT_LoCHO intermediate and greater than HiCHO. HiCHO derived more of its circulating total energy from glucose and triglycerides than did either LoCHO food, while both LoCHO foods derived a larger percentage of total circulating energy from fatty acids. Further, the FAT_LoCHO food had an elevated percentage of circulating energy from βHB relative to either HiCHO or PROT_LoCHO. The current results in canines are in concordance with the aforementioned observations for a human cohort [81]. Levels of circulating leucine (a strictly ketogenic amino acid) and all amino acids that are both ketogenic and glucogenic, which were significantly different between these groups, were decreased following FAT_LoCHO consumption. Thus, an additional source of circulating βHB that must be considered particularly relevant for the FAT_LoCHO food is that body tissue catabolism of proteins increases the availability of ketogenic amino acids. Ketosis acts to preserve lean mass via multiple mechanisms [82]; thus, the difference in apparent decreased amino acid catabolism to generate BUN with the FAT_LoCHO food versus the PROT_LoCHO food may have been influenced by the higher level of βHB achieved with the FAT_LoCHO food and not only the lower protein intake level in this group. 

A model of lipid energy homeostasis has been proposed for humans that posits that reduction in dietary carbohydrate can lead to increased total cholesterol, particularly in individuals who are metabolically fit, active, and near ideal BW [83]. This is proposed to involve a competition between peripheral use of free fatty acids as fuel, hepatic ketone production from the fatty acids, and hepatic re-esterification of the fatty acids to triglycerides. Liver export of re-esterified triglycerides as very low-density lipoproteins requires cholesterol and may underpin the observations of increased lipoprotein-associated cholesterol in humans. This may explain the increased cholesterol on the two LoCHO foods in the current study. A caveat is that only total cholesterol was measured, with no selective assessment of the source of the cholesterol, so there is no information on the relative contribution of very low-, intermediate-, low-, and high-density lipoprotein fractions to the total cholesterol values. Our data are, however, overall supportive of the lipid energy model in canines. The canine subjects enrolled in the current study had clinical blood work values in the normal range, were near or at ideal BW as determined by a colony veterinarian performing body condition scoring, had access to spacious outdoor areas to run, and since they were fed once daily, they spent a large portion of the circadian period in the postabsorptive state. In these dogs, carbohydrate restriction increased circulating total fatty acids, increased total cholesterol, and decreased triglycerides. 

In further support of this model, metabolomics data showed that consumption of the FAT_LoCHO food resulted in higher levels than HiCHO for circulating monoglycerides, acylcarnitines, and β-oxidized fatty acids, all of which are catabolic intermediates of energy harvest from fatty acids. In addition, circulating βHB, a downstream energetic metabolite produced when fatty acid availability exceeds hepatic oxidation capacity, was increased following consumption of the FAT_LoCHO food. In contrast, structural-type lipids, including choline- and ethanolamine-containing phospholipids, were largely decreased with FAT_LoCHO. Hepatic caveolin-1, a caveolar lipid raft protein involved in endocytotic trafficking and intracellular signal transduction, is linked to metabolic switching of glucose and fatty-acid metabolism [84]. Caveolin-1 activity also links together hepatic fatty acid oxidation and bile-acid signaling [85]. Whether changes in caveolin-1 activity or expression underpin the combined results observed here for fat metabolism (βHB, acylcarnitines, fatty acids, triglycerides) and bile-acid levels is a subject for future investigation. Additionally, new research will be required to parse out the various subtypes of lipids within total circulating fatty acids that contribute to circulating energy when dogs consume foods of varying macronutrient makeup. When caloric intake, BW, and circulating energy profiles are taken together, the current study indicates that reduction of dietary carbohydrate by replacement with either protein or fat increases the energy required to maintain BW and that replacement of carbohydrate by fat has a greater effect than replacement by protein in this regard. In addition, postabsorptive energy availability is predominantly from glucose and triglycerides when fed the HiCHO food, gluconeogenic amino acids and fatty acids when fed PROT_LoCHO, and fatty acids as well as βHB when fed FAT_LoCHO.

NAAN, many of which are active at endocannabinoid receptors, are drivers of whole-body metabolism [86] pertinent to weight maintenance [87] through their effects on the endocrine system [88]. Dogs and humans share many traits in common in regard to the endocannabinoid system [89], especially with exercise [90] and obesity [91]. Acylcholines, including those increased by FAT_LoCHO in the current study, are associated with serum lipid markers in humans [92]. Antagonism of the endocannabinoid receptor CB1 increases hepatic insulin sensitivity [93], and CB1 agonists (2-arachidonoylglycerol, palmitoyl ethanolamide) were decreased in the PROT_LoCHO food relative to the HiCHO food in the current study. Obese dogs express increased levels of genes associated with the endocannabinoid pathway, including a lipase that decreases O-acylglycerol endocannabinoid levels [94]. In that report, the authors propose that increased ABDH12 lipase may protect the skeletal muscle system from excess endocannabinoids present in the obese condition [94]. In the current study, the PROT_LoCHO group showed decreased levels of the O-acylglycerol endocannabinoid substrates of the ABDH12 lipase relative to HiCHO, perhaps indicating a role for this food in mitigating adverse effects of obesity on skeletal muscle. The O-acylglycerol endocannabinoid 2-arachidonoylglycerol is increased in circulation of dogs with chronic gastroenteritis relative to healthy dogs [95] and in the synovial fluid of dogs with osteoarthritis [96]. In the current study, 2-arachidonoylglycerol decreased following consumption of both LoCHO foods relative to HiCHO, perhaps offering an opportunity for dietary carbohydrate replacement by protein or fat to ameliorate chronic inflammatory conditions in dogs. Since the PROT_LoCHO food decreased canonical endocannabinoids associated with obesity while FAT_LoCHO mostly increased non-endocannabinoid NAAN, it could be that the apparent increased dietary energy required for weight maintenance was driven to a larger degree by endocannabinoids by the PROT_LoCHO food, but driven more by dietary energy type (e.g., fat) by the FAT_LoCHO food. 

### 3.4. Unique Effect of High Fat, Low Carbohydrate Food to Decrease Indicators of Fatty Acid Desaturase Activity 

Stearoyl-CoA desaturase (SCD1) generates monounsaturated fatty acids (MUFA) from long-chain saturated precursors, while both Δ5 and Δ6 desaturases add additional double bonds to PUFA (here, n3 or n6). A high-fat food has previously been shown to decrease desaturase activity in a rodent model [97]. As well, high carbohydrate intake increases desaturase activity associated with de novo lipogenesis in humans [77]. Increasing provision of carbohydrate to dogs, albeit as sucrose rather than starch (the dietary form from grains fed here), increased fatty acid desaturase activity [98]. Both SCD1 and Δ6 desaturase activities are positively associated in animal models or humans with increased overall mortality [99,100] and cardiovascular mortality [99], obesity-related metabolic comorbidities [101], diabetes incidence [102], and hepatic fibrosis [103]. In addition, the product of SCD1, palmitoleic acid, induces pancreatic lipotoxicity [104]. It should be considered, however, that hepatic and pancreatic fatty acid desaturation by SCD1 may be a protective response to SFA-induced lipotoxicity, since production of MUFA from long-chain SFA can decrease pancreatic cell damage from SFA [105]. Increased SCD1 activity reduces tumor susceptibility to cell death by ferroptosis [106], and reduction of tumor SCD1 activity is a target of anti-cancer therapies [107], since prognosis is worsened for breast and kidney cancers when tumors overexpress SCD1 [108,109]. Low-carbohydrate ketogenic foods have been examined extensively for therapeutic benefit to cancer patients [110]. The canonical mechanism invoked for their use in cancer therapy is the ‘starvation’ of tumors from their preferred energy source, glucose [111]. However, other mechanisms may underpin the effectiveness of ketogenic foods in cancer. A ketogenic food with carbohydrate replaced by fat has been shown to reduce tumor SCD1 activity in a mouse allograft model [112]. The anti-tumorigenic mechanism of action of SCD1 inhibition is via reduction of the cellular ratios of MUFA/SFA (e.g., C16:1/C16:0) [113]. In a mouse allograft model, the type of dietary fat dictated anti-tumorigenicity of the ketogenic food, such that only when MUFA was restricted and SFA increased did the ketogenic food become effective [112]. In the current study, the consumption of the FAT_LoCHO food resulted in ratios of fatty acids that were consistent with decreased SCD1 and Δ6 desaturase activity compared to the HiCHO and PROT_LoCHO foods. It is potentially important that the FAT_LoCHO food decreased both the C16:1/C16:0 and C18:1/C18:0 SCD1 activity indicators since the ketogenic food was only effective when both ratios were decreased in the mouse tumor allograft model [112]. In contrast to SCD1 and Δ6 desaturase, the Δ5 desaturase activity is inversely associated with the aforementioned metabolic conditions (e.g., diabetes) such that some studies have shown a protective effect against disease [101]. However, Δ5 desaturase worsens prognosis for patients with some cancers but not others [114]. In this context, inhibition of Δ5 desaturase has been suggested to provide a therapeutic opportunity in certain cancers [114]. Similar to its effects to decrease SCD1 and Δ6 desaturase, the FAT_LoCHO food also decreased the apparent activity of Δ5 desaturase relative to both HiCHO and PROT_LoCHO foods. 

It may be that the difference in both fat and carbohydrate between the FAT_LoCHO and HiCHO foods, rather than the presence or absence of a single nutrient, led to the stark contrast in desaturase and elongase activities between these foods. Indeed, the PROT_LoCHO food did not lead to a high intake of fat and did not manifest the same desaturase/elongase pattern as the FAT_LoCHO food. It has been proposed that apparent desaturase activity levels may be influenced by dietary fatty acid types [101]. Dietary intake of C18:2n6 and C18:3n3 decrease expression of Δ5 desaturase and Δ6 desaturase in humans [115]. However, six months of feeding of two fats to dogs that contained different amounts of C18:2n6 and C18:3n3 did not alter the activity of liver SCD1 or Δ6 desaturase [116]. In the current study, it does not appear that dietary intake of C18:2n6 was a primary driver as intakes only differed by 5% between the HiCHO and FAT_LoCHO foods, but may have been influenced by intake of C18:3n3 since that was approximately doubled with the consumption of the FAT_LoCHO food relative to HiCHO. The increased intake of C20:5n3 (EPA) and C22:6n3 (DHA) from the FAT_LoCHO food may have also led to decreased Δ5 and Δ6 desaturase activity, as previously shown [117]. However, enzyme product feedback inhibition by dietary fatty acid ratios did not appear to underpin decreased desaturase activity with the FAT_LoCHO food since this intake of dietary ratios of product/substrate (C16:1/C16:0, C18:1/C18:0, C18:3n6/C18:2n6, or C20:4n6/C20:3n6) was not elevated relative to the other two foods. Further, the PROT_LoCHO food had the highest dietary ratio for the Δ6 desaturase product/substrate pair (C18:3n6/C18:2n6), yet, also had the highest Δ6 desaturase activity. It is concluded that differences among the levels and types of fats in the foods in the current study, rather than the carbohydrate energy level alone, contributed to the uniform reduction of apparent desaturase activity in the FAT_LoCHO group. 

Inhibition of Δ5 desaturase by carbohydrate replacement with fat, particularly saturated fat, could possibly provide a therapeutic anti-tumorigenic effect for some cancers. Taken together, this uniform suppression of fatty acid desaturase activity indicators by FAT_LoCHO may offer an opportunity to benefit to aid canines with metabolic diseases or certain cancers.

### 3.5. Consolidated Results Indicate Potential for Improved Nutritional Support for Cancer When Fat, Rather Than Protein, Replaces Carbohydrate

Taken together, the data shown here support a model whereby reduction of digestible carbohydrate in canine foods increases the dietary energy required to maintain BW and increases post-absorptive circulating energy availability, while decreasing the percentage of circulating energy found as glucose. Considering the use of low carbohydrate foods as nutritional support for cancer therapy, it may be that replacement of carbohydrate with fat rather than protein will improve effectiveness by increasing circulating ketones, decreasing the proportion of available energy found as glucose, inhibiting oncogenic desaturases, and shifting the balance of circulating lipids away from structural lipids towards catabolic intermediates. 

## 4. Methods

### 4.1. Study Foods and Analyses

The three foods used in this study were high in carbohydrate (HiCHO, a typical standard adult maintenance food), high in protein but low in carbohydrate (PROT_LoCHO), and high in fat but low in carbohydrate (FAT_LoCHO). Both low carbohydrate foods were formulated with less than 10% energy as carbohydrate, an amount typically considered ketogenic for humans and on par with previous reports of low carbohydrate food formulations for canines [3,32,43,44,54]. Additionally, MCT comprised 18% and long-chain PUFA from fish oil comprised 1% of the total dietary fat in the FAT_LoCHO food, in order to enhance ketogenesis and support adequate n3 PUFA levels (vide infra). Nutrients of the foods were determined by a commercial laboratory (Eurofins Scientific, Inc., Des Moines, IA, USA). Proximate analyses were completed using the following techniques from the Association of Official Agricultural Chemists (AOAC): moisture, AOAC 930.15; protein, AOAC 2001.11; fat, AOAC 954.02; fiber, AOAC 962.09; ash, AOAC 942.0; sugar profile and total sugars, AOAC 977.20; starch, AOAC 996.11 [118]. Fatty acids were measured via gas chromatography of methyl esters [119]. Sugars are a sum of the individual analytic values for fructose, glucose, lactose, maltose, and sucrose. Total dietary fiber is a sum of the analytic values for insoluble and soluble fiber.

The canonical ketogenic ratio (KR) is an empirically derived formula whereby a combination of given proportions of macronutrients can be expected to result in nutritional ketosis in humans [120]. The KR is depicted in Equation (1) [49], where P = protein, F = fat and C = digestible carbohydrate; all in grams per 100 g food as consumed.

KR = (0.46P + 0.9F)/(0.58P + 0.1F + C)(1)

### 4.2. Animals and Experimental Design

Thirty-six beagles (18 neutered males and 18 spayed females) were included in the study. Dogs were excluded from the study if they had been diagnosed with a chronic disease condition, including, but not limited to, kidney disease, hypothyroidism, or diabetes. The mean ± standard deviation age at randomization was 7.1 ± 2.7 years, with body weight (BW) 10.3 ± 1.5 kg. 

In this prospective, randomized crossover trial, dogs consumed the HiCHO food for 4 weeks, with collections in the last week of feeding. Dogs were then randomized to either the PROT_LoCHO or FAT_LoCHO foods, then in a crossover design, switched to the other food they had not yet eaten. Each LoCHO food was fed for 5 weeks, with collections in the fifth week, for a total of 14 weeks of feeding. 

All animals were group housed in pairs at, and maintained by, Hill’s Pet Nutrition Pet Nutrition Center by Hill’s Science and Technology employees and treated in accordance with Hill’s Global Animal Welfare Policy. Prior to and upon completion of the study, each dog received a complete physical exam, blood work, and urinalysis to rule out occult systemic disease. Once enrolled, dogs were fed daily based upon individual metabolic BW, and food intake was recorded. Water was offered ad libitum. Each dog was weighed every week for the duration of the study. All study animals were allowed normal socialization and enrichment activities and the design of the study did not interfere with the animals’ normal daily routine, except that dogs were kept from the outside area for the day prior to fecal collections so as to minimize detritus (e.g., sticks, grass) in the stool samples. Daily group exercise in outdoor grassy runs allowed for exposure to potentially immunogenic environmental and seasonal factors. One dog was removed from the study after a diagnosis of inflammatory bowel disease unrelated to study foods or procedures. All other dogs returned to the colony healthy after the study and no invasive procedures were used. 

All experiments were carried out with approval from the Hill’s Pet Nutrition Institutional Animal Care and Use Committee (IACUC) and in accordance with Hill’s Global Animal Welfare Policy. At no time were animals subjected to any procedures expected to cause pain, discomfort, or distress.

### 4.3. Sample Collection and Analyses

Fasted blood collections were performed under approved IACUC protocols (Protocol #: FP885.1.1.0-A-C-D-ADH-MULTI-112-MULTI). Clinical blood chemistry analysis was performed on a COBAS c501 module (Roche Diagnostics Corporation, Indianapolis, IN, USA). Circulating βHB as a marker of nutritional ketosis was assessed by enzymatic reaction (IDEXX BioAnalytics Inc., Columbia, MO, USA). Serum fatty acids were analyzed as methyl esters by gas chromatography with flame ionization detection. 

Fatty acid elongation and desaturase enzymes were estimated from the relevant lipid ratios [48]. Metabolon (Morrisville, NC, USA) performed the metabolomics analysis of the plasma as previously described [121,122].

Feces were collected as previously described [122] and assessed for adequate stool quality and health parameters. There were no notable findings to report.

A composite of total potential energy availability in the postabsorptive state was approximated from available quantitative circulating markers consisting of glucose (4 kcal/g), βHB (4.2 kcal/g), triglycerides (9 kcal/g), and total fatty acids minus triglycerides (adjusted fatty acids; 9 kcal/g) in a modification of a method used by Shimy et al. [81]. Further, a summation of these four metrics produced a value for total apparent circulating energy with units of kcal/L. Total fatty acids consisted of both NEFA and those derived from complex lipids such as triglycerides, phospholipids, and cholesteryl esters [123]. Since fatty acids from triglycerides would be accounted for by the assay for total fatty acids, the caloric contribution of triglycerides was subtracted to avoid double counting fat energy from triglycerides. 

### 4.4. Statistical Analysis 

Data from the dog removed from the study were not included in the analyses. 

The following statistical analyses were performed in JMP (Version 15.0. SAS Institute Inc., Cary, NC, USA, 1989–2022): multivariate analysis of variance (MANOVA), linear mixed model, dependent samples paired *t*-test, and Huber’s Loss outlier detection. Significance was set at α = 0.05. To assess the degree to which an entire class of of metabolites differed across the diet groups, MANOVA was performed using the Identity function, which fits a model for each metabolite individually, then jointly tests the models together. The separate MANOVA *p* values for Wilks’ Lambda, Pillai’s Trace, Hotelling–Lawley, and Roy’s Max Root are reported in Table S1: Serum Metabolomics Data. Only where *p* values for all of these metrics were less than 0.05 was the multivariate metabolite class considered to be significantly impacted by diet. To assess the overall effect of diet on clinical analytes, fatty acids, and individual members of metabolite classes, univariate linear mixed modeling was performed. In this model, diet was the main effect and subject was the random effect to account for the repeated measures design. Outliers were identified according to the Huber’s Loss function [124] in JMP. Determination of whether individual metabolites differed pairwise between diet treatments, a dependent sample paired *t*-test was performed for each combination of diets on a per-subject basis. 

## Figures and Tables

**Table 1:** Analyzed nutrient content of experimental foods.

Food Component	HiCHO	PROT_LoCHO	FAT_LoCHO
Ketogenic ratio	0.46	0.97	1.63
Metabolizable energy	3353.70	3260.95	4062.45
Carbohydrate, % kcal	38.20	7.66	4.80
Protein, % kcal	24.72	52.92	26.66
Fat, % kcal	37.08	39.41	68.54
Starch	35.50	6.40	4.60
Sugars	1.10	0.74	0.97
Carbohydrate	36.60	7.14	5.57
Protein	23.69	49.31	30.94
Fat	14.63	15.12	32.76
Total dietary fiber	10.60	13.60	14.20
Insoluble fiber	8.50	12.90	12.50
Soluble fiber	2.10	0.70	1.70
SFA	3.60	4.19	13.25
MUFA	5.17	5.59	10.32
n3 PUFA	0.45	0.57	1.77
n6 PUFA	3.61	3.08	4.35
n6/n3 Ratio	8.02	5.40	2.46
C8:0	<0.02	<0.02	3.07
C10:0	<0.02	<0.02	2.93
C12:0	<0.02	<0.02	0.03
C14:0	0.06	0.07	0.20
C16:0	2.76	3.00	5.33
C16:1	0.59	0.67	1.28
C18:0	0.70	1.01	1.57
C18:1	4.48	4.81	8.84
C18:2n6	3.50	2.80	3.97
C18:3n6	<0.02	0.03	0.05
C18:3n3	0.43	0.52	1.36
C20:3n6	<0.02	0.03	0.05
C20:4n6 (ARA)	0.05	0.16	0.19
C20:5n3 (EPA)	<0.02	<0.02	0.18
C22:6n3 (DHA)	<0.02	<0.02	0.14

Values are presented as g/100 g food as fed unless otherwise noted. Sugars are a sum of individual analytical values for fructose, glucose, lactose, maltose, and sucrose. Total dietary fiber is a sum of the analytical values for insoluble and soluble fiber. ARA, arachidonic acid; DHA, docosahexaenoic acid; EPA, eicosapentaenoic acid; MUFA, monounsaturated fatty acids; PUFA, polyunsaturated fatty acids; SFA, saturated fatty acids.

**Table 2:** Intake of dietary nutrients.

Nutrient Intake	HiCHO	PROT_LoCHO	FAT_LoCHO	Mixed Model *p*
Metabolizable energy	94.08 ± 2.92 ^b^	106.20 ± 3.08 ^a^	104.52 ± 3.42 ^a^	0.0004
Starch	9.96 ± 0.31 ^a^	2.09 ± 0.06 ^b^	1.19 ± 0.04 ^c^	<0.0001
Sugars	0.31 ± 0.01 ^a^	0.24 ± 0.01 ^b^	0.25 ± 0.01 ^b^	<0.0001
Carbohydrate	10.27 ± 0.32 ^a^	2.32 ± 0.07 ^b^	1.44 ± 0.05 ^c^	<0.0001
Protein crude	6.65 ± 0.21 ^c^	16.08 ± 0.47 ^a^	7.99 ± 0.26 ^b^	<0.0001
Fat crude	4.11 ± 0.13 ^c^	4.93 ± 0.14 ^b^	8.46 ± 0.28 ^a^	<0.0001
Total dietary fiber	2.97 ± 0.09 ^c^	4.43 ± 0.13 ^a^	3.67 ± 0.12 ^b^	<0.0001
Insoluble fiber	2.39 ± 0.07 ^c^	4.21 ± 0.12 ^a^	3.23 ± 0.11 ^b^	<0.0001
Soluble fiber	0.59 ± 0.02 ^a^	0.23 ± 0.01 ^c^	0.44 ± 0.01 ^b^	<0.0001
MUFA	1.45 ± 0.05 ^c^	1.82 ± 0.05 ^b^	2.66 ± 0.09 ^a^	<0.0001
n3 PUFA	0.13 ± 0.00 ^c^	0.19 ± 0.01 ^b^	0.46 ± 0.01 ^a^	<0.0001
n6 PUFA	1.01 ± 0.03 ^b^	1.00 ± 0.03 ^b^	1.12 ± 0.04 ^a^	0.0006
C8:0	<0.006	<0.007	0.79 ± 0.03	NA
C10:0	<0.006	<0.007	0.76 ± 0.02	NA
C12:0	<0.006	<0.007	0.01 ± 0.00	NA
C14:0	0.02 ± 0.00 ^c^	0.02 ± 0.00 ^b^	0.05 ± 0.00 ^a^	<0.0001
C16:0	0.77 ± 0.02 ^c^	0.98 ± 0.03 ^b^	1.38 ± 0.05 ^a^	<0.0001
C16:1	0.17 ± 0.01 ^c^	0.22 ± 0.01 ^b^	0.33 ± 0.01 ^a^	<0.0001
C18:0	0.20 ± 0.01 ^c^	0.33 ± 0.01 ^b^	0.41 ± 0.01 ^a^	<0.0001
C18:1	1.26 ± 0.04 ^c^	1.57 ± 0.05 ^b^	2.28 ± 0.08 ^a^	<0.0001
C18:2n6	0.98 ± 0.03 ^a,b^	0.91 ± 0.03 ^b^	1.03 ± 0.03 ^a^	0.0015
C18:3n6	<0.006	<0.010	0.01 ± 0.00	NA
C18:3n3	0.12 ± 0.00 ^c^	0.17 ± 0.01 ^b^	0.35 ± 0.01 ^a^	<0.0001
C20:3n6	< 0.006	<0.010	0.01 ± 0.00	NA
C20:4n6 (ARA)	0.01 ± 0.00 ^b^	0.05 ± 0.00 ^a^	0.05 ± 0.00 ^a^	<0.0001
C20:5n3 (EPA)	<0.006	<0.007	0.05 ± 0.00	NA
C22:6n3 (DHA)	<0.006	<0.007	0.04 ± 0.00	NA

Values other than metabolizable energy are presented as mean ± standard error of daily g intake/(kg BW^0.75^). Metabolizable energy was calculated by Atwater equation using the sum of total sugars and starch for carbohydrate; values presented as mean ± standard error of daily kcal intake/(kg BW^0.75^). The term Sugars designates a sum of individual analytical values for fructose, glucose, lactose, maltose, and sucrose. The term Carbohydrate designates a sum of sugars and starch. The term Total dietary fiber indicates a sum of the analytical values for insoluble and soluble fiber. Superscript letters differing within a row across foods denote means that are significantly different at *p* < 0.05 by dependent samples paired *t*-test. NA is used to designate those comparisons in which a nutrient was below the limit of quantitation. ARA, arachidonic acid; BW, body weight; DHA, docosahexaenoic acid; EPA, eicosapentaenoic acid; MUFA, monounsaturated fatty acids; NA, not applicable; PUFA, polyunsaturated fatty acids; SFA, saturated fatty acids.

**Table 3:** Serum clinical analytes.

Analyte	HiCHO	PROT_LoCHO	FAT_LoCHO	Mixed Model *p*
Albumin (g/dL)	3.44 ± 0.05 ^c^	3.62 ± 0.04 ^a^	3.52 ± 0.05 ^b^	<0.0001
BUN (mg/dL)	11.71 ± 0.40 ^b^	18.26 ± 0.65 ^a^	12.49 ± 0.49 ^b^	<0.0001
Creatinine (mg/dL)	0.65 ± 0.02 ^b^	0.69 ± 0.02 ^a^	0.67 ± 0.02 ^a,b^	0.004
Bilirubin, total (mg/dL)	0.09 ± 0.01	0.08 ± 0.01	0.07 ± 0.007	0.146
Glucose (mM)	5.27 ± 0.07	5.22 ± 0.09	5.23 ± 0.10	0.838
β-hydroxybutyrate (uM)	93.59 ± 2.96 ^b^	101.27 ± 6.24 ^b^	143.81 ± 13.79 ^a^	<0.0001
Triglycerides (mM)	0.62 ± 0.03 ^a^	0.43 ± 0.02 ^b^	0.43 ± 0.01 ^b^	<0.0001
Cholesterol (mM)	4.83 ± 0.18 ^c^	5.55 ± 0.20 ^b^	6.37 ± 0.20 ^a^	<0.0001
Lipemic index (mg/dL)	5.00 ± 0.31	4.91 ± 0.36	4.69 ± 0.27	0.758

Superscript letters differing within a row across foods denote means that are significantly different at *p* < 0.05 by dependent samples paired *t*-test. BUN, blood urea nitrogen.

**Table 4:** Effects of foods on serum amino acids.

Type	Amino Acid	Overall Food Effect	Protein Replaces CHO	Fat Replaces CHO	Fat Instead of Protein Replacing CHO
Catabolite	urea				
Ketogenic Only	leucine				
	lysine				
Ketogenic and Glucogenic	isoleucine				
	phenylalanine				
	threonine				
	tryptophan				
	tyrosine				
Glucogenic Only	alanine				
	arginine				
	asparagine				
	aspartate				
	cysteine				
	cystine				
	glutamate				
	glutamine				
	glycine				
	histidine				
	methionine				
	proline				
	serine				
	valine				
Other	taurine				

Significant main effects of food (*p* < 0.05) for a metabolite by linear mixed model are shown as yellow-filled cells. Significant differences (*p* < 0.05) between levels of a metabolite by dependent samples paired *t*-test for all pairwise food comparisons are shown as filled cells where red-filled cells are positive values and green are negative values. Protein Replaces CHO = PROT_LoCHO—HiCHO. Fat Replaces CHO = FAT_LoCHO—HiCHO. Fat Rather than Protein Replacing CHO = FAT_LoCHO—PROT_LoCHO. Full numerical data are available in Table S1.

**Table 5:** Effects of foods on serum kynurenine pathway metabolites.

Kynurenine Pathway Metabolites	Overall Food Effect	Protein Replaces CHO	Fat Replaces CHO	Fat Instead of Protein Replacing CHO
kynurenate				
kynurenine				
N-acetylkynurenine				
N-formylkynurenine				
anthranilate				
N-formylanthranilic acid				
quinolinate				
picolinate				
1-methylnicotinamide				
nicotinamide				
nicotinamide riboside				
N′-methylnicotinate				

Significant main effects of food (*p* < 0.05) for a metabolite by linear mixed model are shown as yellow-filled cells. Significant differences (*p* < 0.05) between levels of a metabolite by dependent samples paired *t*-test for all pairwise food comparisons are shown as filled cells where red-filled cells are positive values and green are negative values. Protein Replaces CHO = PROT_LoCHO—HiCHO. Fat Replaces CHO = FAT_LoCHO—HiCHO. Fat Rather than Protein Replacing CHO = FAT_LoCHO—PROT_LoCHO. Full numerical data are available in Table S1.

**Table 6:** Serum total fatty acids.

Analyte	HiCHO	PROT_LoCHO	FAT_LoCHO	Mixed Model *p*
C12:0	6.20 ± 0.52 ^a^	2.36 ± 0.53 ^b^	3.60 ± 0.58 ^b^	<0.0001
C14:0	22.50 ± 1.06 ^a^	16.83 ± 1.04 ^b^	14.31 ± 0.75 ^c^	<0.0001
C16:0	1336.32 ± 30.34 ^b^	1493.36 ± 33.71 ^a^	1398.01 ± 27.66 ^b^	<0.0001
C16:1	99.91 ± 4.20 ^a^	94.65 ± 4.22 ^a^	78.56 ± 2.97 ^b^	<0.0001
C18:0	2255.08 ± 71.00 ^c^	2435.94 ± 65.71 ^b^	2975.93 ± 75.71 ^a^	<0.0001
C18:1	843.22 ± 22.63 ^a^	813.09 ± 24.28 ^a,b^	795.71 ± 20.19 ^b^	0.0509
C18:2n6	1763.02 ± 44.48 ^a^	1554.49 ± 35.22 ^c^	1634.83 ± 36.17 ^b^	<0.0001
C18:3n3	53.36 ± 1.56 ^b^	65.94 ± 2.24 ^a^	69.09 ± 2.59 ^a^	<0.0001
C18:3n6	6.67 ± 0.24 ^a^	6.23 ± 0.26 ^a^	4.60 ± 0.26 ^b^	<0.0001
C20:2n6	23.45 ± 1.08 ^a^	18.92 ± 0.72 ^b^	18.08 ± 0.80 ^b^	<0.0001
C20:3n6	103.25 ± 6.54 ^b^	105.98 ± 6.00 ^b^	155.01 ± 6.38 ^a^	<0.0001
C20:4n6 (ARA)	1769.17 ± 59.31 ^b^	2199.47 ± 59.25 ^a^	2146.05 ± 64.18 ^a^	<0.0001
C20:5n3 (EPA)	23.31 ± 1.13 ^b^	35.65 ± 1.23 ^b^	164.29 ± 6.84 ^a^	<0.0001
C22:4n6	78.60 ± 3.23 ^b^	119.88 ± 3.76 ^a^	43.36 ± 1.32 ^c^	<0.0001
C22:5n3	220.93 ± 9.30 ^b^	196.78 ± 8.50 ^c^	254.16 ± 8.70 ^a^	<0.0001
C22:6n3 (DHA)	100.08 ± 5.62 ^b^	89.43 ± 3.95 ^b^	258.80 ± 8.67 ^a^	<0.0001
Total FA (mM)	8.71 ± 0.23 ^c^	9.25 ± 0.21 ^b^	10.0 ± 0.22 ^a^	<0.0001
n6/n3 (mol ratio)	9.63 ± 0.25 ^b^	10.43 ± 0.19 ^a^	5.40 ± 0.12 ^c^	<0.0001
SFA/UFA (mol ratio)	0.71 ± 0.01 ^c^	0.74 ± 0.01 ^b^	0.78 ± 0.01 ^a^	<0.0001
Total MUFA (mol %)	10.92 ± 0.27 ^a^	9.85 ± 0.27 ^b^	8.76 ± 0.17 ^c^	<0.0001
Total n3 (mol %)	4.55 ± 0.11 ^b^	4.20 ± 0.08 ^c^	7.48 ± 0.12 ^a^	<0.0001
Total n6 (mol %)	42.98 ± 0.18 ^a^	43.29 ± 0.14 ^a^	39.91 ± 0.21 ^b^	<0.0001
Total PUFA (mol %)	47.53 ± 0.18	47.49 ± 0.15	47.39 ± 0.12	0.7432
Total SFA (mol %)	41.55 ± 0.17 ^c^	42.66 ± 0.18 ^b^	43.85 ± 0.16 ^a^	<0.0001
Total UFA (mol %)	58.45 ± 0.17 ^a^	57.34 ± 0.18 ^b^	56.15 ± 0.16 ^c^	<0.0001

Mixed Model *p* indicates significant main effects of food (*p* < 0.05) for a fatty acid or fatty acid aggregate value. Significant differences (*p* < 0.05) between levels of a fatty acid by dependent samples paired *t*-test for all pairwise food comparisons are shown as differing superscript letters. Units of measure are μM unless otherwise noted. ARA, arachidonic acid; DHA, docosahexaenoic acid; EPA, eicosapentaenoic acid; MUFA, monounsaturated fatty acids; PUFA, polyunsaturated fatty acids; SFA, saturated fatty acids; UFA, unsaturated fatty acids.

**Table 7:** Elongation and desaturation fatty acid ratios.

Fatty Acid Ratios	HiCHO	PROT_LoCHO	FAT_LoCHO	Mixed Model *p*
SCD1 (Δ9) (16:1/16:0)	0.08 ± 0.00 ^a^	0.06 ± 0.00 ^b^	0.06 ± 0.00 ^c^	<0.0001
SCD1 (Δ9) (18:1/18:0)	0.38 ± 0.01 ^a^	0.34 ± 0.01 ^b^	0.27 ± 0.01 ^c^	<0.0001
Δ6 desaturase (18:3n6/18:2n6)	0.004 ± 0.00 ^a^	0.004 ± 0.00 ^a^	0.003 ± 0.00 ^b^	<0.0001
Δ5 desaturase (20:4n6/20:3n6)	18.92 ± 1.10 ^b^	22.02 ± 0.87 ^a^	14.29 ± 0.51 ^c^	<0.0001
Elongase Elovl-6 (18:0/16:0)	1.68 ± 0.03 ^b^	1.63 ± 0.03 ^b^	2.13 ± 0.03 ^a^	<0.0001
Elongase Elovl-5 (20:3n6/18:3n6)	16.37 ± 1.34 ^b^	18.06 ± 1.21 ^b^	37.18 ± 2.57 ^a^	<0.0001
Overall Elongation ((18:0 + 18:1)/16:0)	2.32 ± 0.03 ^b^	2.18 ± 0.02 ^c^	2.70 ± 0.03 ^a^	<0.0001

Mixed Model *p* indicates significant main effects of food (*p* < 0.05) for a metabolite. Significant differences (*p* < 0.05) between levels of a metabolite by dependent samples paired *t*-test for all pairwise food comparisons are shown as differing superscript letters. Units of measure are ratios of fatty acids μM/μM. Elovl, elongation of very long chain fatty acids; SCD1, stearoyl CoA desaturase.

**Table 8:** Serum total apparent circulating energy and constituents thereof.

Analyte	HiCHO	PROT_LoCHO	FAT_LoCHO	Mixed Model *p*
Apparent Circulating Energy ^1^	24.99 ± 0.56 ^c^	26.29 ± 0.52 ^b^	28.12 ± 0.53 ^a^	<0.0001
Glucose (% of energy)	15.41 ± 0.37 ^a^	14.48 ± 0.32 ^b^	13.57 ± 0.36 ^c^	<0.0001
βHB (% of energy)	0.17 ± 0.01 ^b^	0.17 ± 0.01 ^b^	0.23 ± 0.02 ^a^	0.0035
Triglycerides (% of energy)	20.06 ± 1.15 ^a^	13.12 ± 0.45 ^b^	12.26 ± 0.35 ^b^	<0.0001
Adjusted Fatty Acids ^2^ (% of energy)	64.36 ± 1.29 ^b^	72.22 ± 0.60 ^a^	73.94 ± 0.52 ^a^	<0.0001

Superscript letters differing within a row across foods denote means that are significantly different at *p* < 0.05 by dependent samples paired *t*-test. ^1^ Apparent circulating energy (kcal/L) = glucose energy (kcal/L) + βHB energy (kcal/L) + triglycerides energy (kcal/L) + adjusted fatty acids energy (kcal/L). ^2^ Adjusted fatty acids = total fatty acids (kcal/L)—triglycerides energy (kcal/L). βHB, beta-hydroxybutyrate.

**Table 9:** Effects of foods on serum β-hydroxy fatty acids and ω−carboxy dioates.

Type	Fatty Acid	Overall Food Effect	Protein Replaces CHO	Fat Replaces CHO	Fat Instead of Protein Replacing CHO
β-hydroxy fatty acid	3-hydroxybutyrate (BHB)				
	3-hydroxyhexanoate				
	3-hydroxyadipate				
	3-hydroxyoctanoate				
	3-hydroxydecanoate				
	3-hydroxysebacate				
	3-hydroxyundecanedioate				
	3-hydroxydodecanedioate				
	3-hydroxylaurate				
	3-hydroxymyristate				
	3-hydroxystearate				
ω-carboxy fatty acid	oxalate (ethanedioate)				
	glutarate (C5-DC)				
	pimelate (C7-DC)				
	heptenedioate (C7:1-DC)				
	suberate (C8-DC)				
	azelate (C9-DC)				
	sebacate (C10-DC)				
	undecanedioate (C11-DC)				
	tridecenedioate (C13:1-DC)				
	tetradecanedioate (C14)				
	hexadecanedioate (C16)				
	hromatographye (C16:1-DC)				
	heptadecanedioate (C17-DC)				
	octadecanedioate (C18)				
	octadecenedioate (C18:1-DC)				
	octadecadienedioate (C18:2-DC)				
	nonadecanedioate (C19-DC)				
	eicosanedioate (C20-DC)				
	eicosenedioate (C20:1-DC)				

Significant main effects of food (*p* < 0.05) for a metabolite by linear mixed model are shown as yellow-filled cells. Significant differences (*p* < 0.05) between levels of a metabolite by dependent samples paired *t*-test for all pairwise food comparisons are shown as filled cells where red-filled cells are positive values and green are negative values. Protein Replaces CHO = PROT_LoCHO—HiCHO. Fat Replaces CHO = FAT_LoCHO—HiCHO. Fat Rather than Protein Replacing CHO = FAT_LoCHO—PROT_LoCHO. Full numerical data are available in Table S1.

**Table 10:** Effects of foods on serum carnitines.

Type	Carnitine	Overall Food Effect	Protein Replaces CHO	Fat Replaces CHO	Fat Instead of Protein Replacing CHO
Carnitine	carnitine				
	deoxycarnitine				
Acylcarnitine	acetylcarnitine (C2)				
	propionylcarnitine (C3)				
	malonylcarnitine				
	butyrylcarnitine (C4)				
	butenoylcarnitine (C4:1)				
	(S)-3-hydroxybutyrylcarnitine				
	I-3-hydroxybutyrylcarnitine				
	succinylcarnitine (C4-DC)				
	glutarylcarnitine (C5-DC)				
	hexanoylcarnitine (C6)				
	adipoylcarnitine (C6-DC)				
	cis-3,4-methyleneheptanoylcarnitine				
	octanoylcarnitine (C8)				
	3-hydroxyoctanoylcarnitine				
	suberoylcarnitine (C8-DC)				
	decanoylcarnitine (C10)				
	cis-4-decenoylcarnitine (C10:1)				
	3-hydroxydecanoylcarnitine				
	undecenoylcarnitine (C11:1)				
	laurylcarnitine (C12)				
	5-dodecenoylcarnitine (C12:1)				
	myristoleoylcarnitine (C14:1)				
	myristoylcarnitine (C14)				
	pentadecanoylcarnitine (C15)				
	palmitoylcarnitine (C16)				
	palmitoleoylcarnitine (C16:1)				
	3-hydroxypalmitoylcarnitine				
	margaroylcarnitine (C17)				
	stearoylcarnitine (C18)				
	octadecanedioylcarnitine (C18-DC)				
	oleoylcarnitine (C18:1)				
	3-hydroxyoleoylcarnitine				
	linoleoylcarnitine (C18:2)				
	linolenoylcarnitine (C18:3)				
	eicosenoylcarnitine (C20:1)				
	dihomo-linoleoylcarnitine (C20:2)				
	dihomo-linolenoylcarnitine (C20:3n3 or 6)				
	arachidoylcarnitine (C20)				
	arachidonoylcarnitine (C20:4)				
	behenoylcarnitine (C22)				
	erucoylcarnitine (C22:1)				
	docosapentaenoylcarnitine (C22:5n3)				
	docosahexaenoylcarnitine (C22:6)				
	lignoceroylcarnitine (C24)				
	nervonoylcarnitine (C24:1)				
	ximenoylcarnitine (C26:1)				
	cerotoylcarnitine (C26)				

Significant main effects of food (*p* < 0.05) for a metabolite by linear mixed model are shown as yellow-filled cells. Significant differences (*p* < 0.05) between levels of a metabolite by dependent samples paired *t*-test for all pairwise food comparisons are shown as filled cells where red-filled cells are increases and green are decreases. Protein Replaces CHO = PROT_LoCHO—HiCHO. Fat Replaces CHO = FAT_LoCHO—HiCHO. Fat Rather than Protein Replacing CHO = FAT_LoCHO—PROT_LoCHO. Full numerical data are available in Table S1.

**Table 11:** Effects of foods on serum choline-containing phospholipids.

Type	Choline-Containing Phospholipid	Overall Food Effect	Protein Replaces CHO	Fat Replaces CHO	Fat Instead of Protein Replacing CHO
Precursor/ Catabolite	choline				
	glycerophosphorylcholine (GPC)				
Lysolipid (LysC)	1-palmitoyl-GPC (16:0)				
	1-palmitoleoyl-GPC (16:1)				
	1-stearoyl-GPC (18:0)				
	1-oleoyl-GPC (18:1)				
	1-linoleoyl-GPC (18:2)				
	1-linolenoyl-GPC (18:3)				
	1-arachidonoyl-GPC (20:4)				
	1-lignoceroyl-GPC (24:0)				
	1-cerotoyl-GPC (26:0)				
	1-(1-enyl-palmitoyl)-GPC (P-16:0)				
Phospholipid (PtdC)	1-myristoyl-2-palmitoyl-GPC (14:0/16:0)				
	1-myristoyl-2-arachidonoyl-GPC (14:0/20:4)				
	1,2-dipalmitoyl-GPC (16:0/16:0)				
	1-palmitoyl-2-palmitoleoyl-GPC (16:0/16:1)				
	1-palmitoyl-2-stearoyl-GPC (16:0/18:0)				
	1-palmitoyl-2-oleoyl-GPC (16:0/18:1)				
	1-palmitoyl-2-linoleoyl-GPC (16:0/18:2)				
	1-palmitoyl-2-arachidonoyl-GPC (16:0/20:4n6)				
	1-palmitoyl-2-docosahexaenoyl-GPC (16:0/22:6)				
	1-(1-enyl-palmitoyl)-2-palmitoyl-GPC (P-16:0/16:0)				
	1-(1-enyl-palmitoyl)-2-palmitoleoyl-GPC (P-16:0/16:1)				
	1-(1-enyl-palmitoyl)-2-oleoyl-GPC (P-16:0/18:1)				
	1-(1-enyl-palmitoyl)-2-linoleoyl-GPC (P-16:0/18:2)				
	1-(1-enyl-palmitoyl)-2-arachidonoyl-GPC (P-16:0/20:4)				
	1-palmitoleoyl-2-linoleoyl-GPC (16:1/18:2)				
	1-palmitoleoyl-2-linolenoyl-GPC (16:1/18:3)				
	1-stearoyl-2-oleoyl-GPC (18:0/18:1)				
	1-stearoyl-2-linoleoyl-GPC (18:0/18:2)				
	1-stearoyl-2-arachidonoyl-GPC (18:0/20:4)				
	1-stearoyl-2-docosahexaenoyl-GPC (18:0/22:6)				
	1-oleoyl-2-docosahexaenoyl-GPC (18:1/22:6)				
	1,2-dilinoleoyl-GPC (18:2/18:2)				
	1-linoleoyl-2-linolenoyl-GPC (18:2/18:3)				
	1-linoleoyl-2-arachidonoyl-GPC (18:2/20:4n6)				
	1,2-dilinolenoyl-GPC (18:3/18:3)				

Significant main effects of food (*p* < 0.05) for a metabolite by linear mixed model are shown as yellow-filled cells. Significant differences (*p* < 0.05) between levels of a metabolite by dependent samples paired *t*-test for all pairwise food comparisons are shown as filled cells where red-filled cells are increases and green are decreases. Protein Replaces CHO = PROT_LoCHO—HiCHO. Fat Replaces CHO = FAT_LoCHO—HiCHO. Fat Rather than Protein Replacing CHO = FAT_LoCHO—PROT_LoCHO.Full numerical data are available in Table S1.

**Table 12:** Effects of foods on serum ethanolamine-containing phospholipids.

Type	Ethanolamine-Containing Phospholipid	Overall Food Effect	Protein Replaces CHO	Fat Replaces CHO	Fat Instead of Protein Replacing CHO
Precursor/ Catabolite	phosphoethanolamine				
	glycerophosphoethanolamine (GPE)				
Lysolipid (LysE)	1-palmitoyl-GPE (16:0)				
	1-stearoyl-GPE (18:0)				
	1-oleoyl-GPE (18:1)				
	1-linoleoyl-GPE (18:2)				
	1-arachidonoyl-GPE (20:4n6)				
	1-(1-enyl-palmitoyl)-GPE (P-16:0)				
	1-(1-enyl-stearoyl)-GPE (P-18:0)				
	1-(1-enyl-oleoyl)-GPE (P-18:1)				
Phospholipid (PtdE)	1-palmitoyl-2-oleoyl-GPE (16:0/18:1)				
	1-palmitoyl-2-linoleoyl-GPE (16:0/18:2)				
	1-palmitoyl-2-arachidonoyl-GPE (16:0/20:4)				
	1-palmitoyl-2-docosahexaenoyl-GPE (16:0/22:6)				
	1-(1-enyl-palmitoyl)-2-oleoyl-GPE (P-16:0/18:1)				
	1-(1-enyl-palmitoyl)-2-linoleoyl-GPE (P-16:0/18:2)				
	1-(1-enyl-palmitoyl)-2-arachidonoyl-GPE (P-16:0/20:4)				
	1-stearoyl-2-oleoyl-GPE (18:0/18:1)				
	1-stearoyl-2-linoleoyl-GPE (18:0/18:2)				
	1-stearoyl-2-arachidonoyl-GPE (18:0/20:4)				
	1-stearoyl-2-docosahexaenoyl-GPE (18:0/22:6)				
	1-(1-enyl-stearoyl)-2-oleoyl-GPE (P-18:0/18:1)				
	1-(1-enyl-stearoyl)-2-linoleoyl-GPE (P-18:0/18:2)				
	1-(1-enyl-stearoyl)-2-arachidonoyl-GPE (P-18:0/20:4)				
	1-oleoyl-2-arachidonoyl-GPE (18:1/20:4)				
	1,2-dilinoleoyl-GPE (18:2/18:2)				

Significant main effects of food (*p* < 0.05) for a metabolite by linear mixed model are shown as yellow-filled cells. Significant differences (*p* < 0.05) between levels of a metabolite by dependent samples paired *t*-test for all pairwise food comparisons are shown as filled cells where red-filled cells are increases and green are decreases. Protein Replaces CHO = PROT_LoCHO—HiCHO. Fat Replaces CHO = FAT_LoCHO—HiCHO. Fat Rather than Protein Replacing CHO = FAT_LoCHO—PROT_LoCHO. Full numerical data are available in Table S1. GPE, glycerophosphorylethanolamine.

**Table 13:** Effects of foods on serum endocannabinoids and acylated neurotransmitters or amino acids.

Type	Endocannabinoids and Acyl Neurotransmitters	Overall Food Effect	Protein Replaces CHO	Fat Replaces CHO	Fat Instead of Protein Replacing CHO
ethanolamide	palmitoyl ethanolamide				
	oleoyl ethanolamide				
acylglycerol	2-oleoylglycerol				
	2-linoleoylglycerol				
	2-arachidonoylglycerol				
acylcholine	palmitoylcholine				
	margaroylcholine				
	stearoylcholine				
	oleoylcholine				
	linoleoylcholine				
	arachidonoylcholine				
	eicosapentaenoylcholine				
	docosahexaenoylcholine				
acyltaurine	hexanoyltaurine				
	N-palmitoyltaurine				
	N-stearoyltaurine				
	N-oleoyltaurine				
	N-linoleoyltaurine				
acylserine	N-stearoylserine				
	N-oleoylserine				
acylglycine	N-palmitoylglycine				

Significant main effects of food (*p* < 0.05) for a metabolite by linear mixed model are shown as yellow-filled cells. Significant differences (*p* < 0.05) between levels of a metabolite by dependent samples paired *t*-test for all pairwise food comparisons are shown as filled cells where red-filled cells are increases and green are decreases. Protein Replaces CHO = PROT_LoCHO—HiCHO. Fat Replaces CHO = FAT_LoCHO—HiCHO. Fat Rather than Protein Replacing CHO = FAT_LoCHO—PROT_LoCHO.Full numerical data are available in Table S1.

**Table 14:** Effects of foods on serum bile acids.

Type	Bile Acid	Overall Food Effect	Protein Replaces CHO	Fat Replaces CHO	Fat Rather Than Protein Replacing CHO
Primary	beta-muricholate				
	cholate				
	cholate glucuronide				
	tauro-beta-muricholate				
	taurocholate				
	ursocholate				
Secondary	12-dehydrocholate				
	12-ketolithocholate				
	3-dehydrocholate				
	7-ketodeoxycholate				
	lithocholate				
	taurodeoxycholate				
	taurohyodeoxycholate				
	taurolithocholate				
	tauroursodeoxycholate				
	ursodeoxycholate				

Significant main effects of food (*p* < 0.05) for a metabolite by linear mixed model are shown as yellow-filled cells. Significant differences (*p* < 0.05) between levels of a metabolite by dependent samples paired *t*-test for all pairwise food comparisons are shown as filled cells where red-filled cells are increases and green are decreases. Protein Replaces CHO = PROT_LoCHO—HiCHO. Fat Replaces CHO = FAT_LoCHO—HiCHO. Fat Rather than Protein Replacing CHO = FAT_LoCHO—PROT_LoCHO. Full numerical data are available in Table S1.

## Data Availability

Study data are available in Table S1.

## Supplementary Materials

The following supporting information can be downloaded at: https://www.mdpi.com/article/10.3390/metabo12070591/s1, Table S1: Serum Metabolomics Data.
